# The Association Between Schoolwork Pressure and Overweight/Obesity in Swedish Schoolchildren Across Different Socioeconomic Groups

**DOI:** 10.1111/ijpo.70067

**Published:** 2025-11-12

**Authors:** Cassandra Comey, Kenisha Russell Jonsson, Maria Corell

**Affiliations:** ^1^ School of Public Health and Community Medicine Sahlgrenska Academy University of Gothenburg Gothenburg Sweden

**Keywords:** children and adolescents, obesity, overweight, schoolwork pressure, socioeconomic status

## Abstract

**Background:**

In Sweden, schoolwork pressure and adolescent overweight/obesity prevalence have increased in recent decades. This study investigates the relationship between schoolwork pressure and weight status, considering the moderating role of socioeconomic background.

**Methods:**

We analysed pooled cross‐sectional data from 12 154 adolescents in the nationally representative Swedish Health Behaviour in School‐aged Children surveys (2013/14, 2017/18, 2021/22). Logistic regression models examined the association between schoolwork pressure and self‐reported overweight/obesity status (standardised by IOTF cut‐offs) across socioeconomic backgrounds assessed by the Family Affluence Scale (FAS) and perceived family wealth (PFW). We calculated population attributable fractions to quantify schoolwork pressure's contribution to overweight/obesity.

**Results:**

37% of students reported high schoolwork pressure, and 14% had overweight or obesity. High schoolwork pressure was associated with increased odds of overweight/obesity (OR 1.17, 95% CI 1.03–1.32). Adolescents experiencing high pressure from low socioeconomic backgrounds FAS (OR 2.10, 95% CI 1.60–2.76) or PFW (OR 1.82, 95% CI 1.22–2.71) had the highest odds of overweight/obesity. Approximately 5% of overweight/obesity cases were attributable to high schoolwork pressure.

**Conclusions:**

High schoolwork pressure increases the likelihood of adolescent overweight/obesity, particularly for disadvantaged socioeconomic groups. Implementing policies to reduce academic stress and providing targeted support are essential for promoting healthier outcomes.

## 1 Introduction

Stress in childhood and adolescence has many sources, including family dynamics, peer interactions, social media exposure and particularly school‐related pressures [1]. Academic demands, such as the volume of assignments, frequent testing, grades, peer relationships, and concerns about the future, can be especially challenging. The Swedish school reform of 2011 introduced increased testing, earlier grading, a new grading system (A‐F), and stricter entrance requirements to high school programmes [2]. Over the past decade, perceived schoolwork pressure or school‐related stress, has steadily increased among adolescents in Sweden, with rising levels observed by age and gender. Girls have consistently reported higher stress levels than boys [3]. In 2021/22, schoolwork pressure was reported by 28% of 11‐year‐old girls, 63% of 13‐year‐old girls, and 78% of 15‐year‐old girls. Among boys, 20% of 11‐year‐olds, 40% of 13‐year‐olds, and 51% of 15‐year‐olds felt some or a lot of stress from schoolwork during the same period. In terms of stress in relation to school, school performance emerged as the most frequently reported stressor among boys and girls across all age groups [3].

Previous research has indicated a correlation between schoolwork pressure and psychological or psychosomatic health complaints cross‐nationally [3, 4] and in Sweden [5, 6]. In addition to psychological and somatic complaints, stress exposure during childhood has been linked to the risk of overweight and obesity [7, 8, 9]. High stress, may trigger obesogenic behaviours such as eating in the absence of hunger and emotional overeating [10]. Even among children as young as 8 years old, stress has been associated with greater consumption of unhealthy foods [11]. These stress‐related dietary patterns, often characterised by an increased intake of sugar, fat, and processed foods, may contribute to weight gain and obesity risk [12].

Additionally, the home environment may influence the relationship between stress and overweight/obesity. Children in single‐parent households may experience greater stress levels [13, 14] and a higher likelihood of overweight and obesity [15, 16] than those in nuclear families. This disparity may stem from differences in financial resources to support healthy behaviours, such as purchasing nutritious food or having access to opportunities for physical activity [16]. Notably, previous research has suggested the reverse relationship exists, where overweight/obesity is associated with significant changes in stress biology over time, from early to middle childhood [17]. The relationship between specifically schoolwork pressure and overweight/obesity in children and adolescents has been researched to a lesser extent, but those studies that exist show that greater levels of school‐related stress are associated with higher odds of overweight and obesity [12, 18].

In Sweden, as with schoolwork pressure, the prevalence of overweight and obesity has shown an upward trend over time, with distinct patterns observed across age and gender groups. Since 1989/90, the prevalence of obesity has tripled among girls and increased fivefold among boys. The proportion of overweight increases gradually with age (11, 13, and 15 years) among boys, while it remains relatively stable among girls [3]. Childhood overweight and obesity are significant public health concerns, partly because of their association with a wide range of physical health complications. These include increased risk of cardiovascular disease and adult morbidity [19], early onset of type II diabetes [20], and a greater prevalence of asthma, allergies, joint problems, and headaches [21] compared to children with normal weight. Besides physical health concerns, childhood obesity appears to be associated with adverse mental health outcomes [22, 23]. A systematic review and meta‐analysis found a positive association between childhood obesity and depression, with a greater risk for girls than boys [22]. In Sweden, a nationwide study also found that children with obesity have a greater risk of depression and anxiety compared to children from the general population [23].

Furthermore, health issues related to overweight and obesity impose financial and social burdens on both society and the individuals affected. A 2022 study by the Swedish Institute for Health Economics (IHE) revealed that childhood overweight and obesity led to higher costs in health care and social services over the life course compared to living with normal weight [24]. The IHE estimated the societal costs of having obesity over a lifetime to be roughly SEK 1.8 billion for 6‐year‐olds and SEK 2.9 billion for 15‐year‐olds in Sweden. Due to its higher prevalence, overweight incurred even greater national costs, SEK 2.1 billion and SEK 4.2 billion, respectively. The study highlighted that childhood obesity incurs greater costs than overweight on an individual level because obesity is more likely to persist into adulthood and is associated with more severe health complications. The IHE primarily attributes the total societal costs to productivity loss due to sick leave in adulthood, noting that the expenses were higher for girls than boys [24].

International research suggests a socioeconomic gradient in the prevalence of childhood overweight and obesity, where socioeconomic background is inversely associated with weight status [25, 26, 27]. Importantly, the association appears to vary depending on the measure of socioeconomic status used (e.g., parental education, parental income, and perceived family wealth). A Swedish study found that for 6‐year‐old boys and girls, being from less affluent families nearly doubled their likelihood of having overweight or obesity as compared to children from higher socioeconomic backgrounds [28].

### 1.1 Theoretical Framework

This study employs the Social Ecological Model (SEM) [29] and Hemmingsson's theoretical model of childhood obesity (2014) [30] to investigate how schoolwork pressure, along with individual and interpersonal factors, correlates with adolescent overweight and obesity. The SEM recognises that individuals are affected by multiple levels of the social and environmental context in which they live. It emphasises how interactions between individuals and their social environment, including family, peers, schools, policies, and socioeconomic inequalities, shape health outcomes [29]. Guided by this framework, this study considers schoolwork pressure, age, gender, and family structure as key variables influencing adolescent weight status at the individual and interpersonal level. Hemmingsson's model highlights how socioeconomic disadvantage leads to psychological distress within families, fostering maladaptive coping strategies in children in response to stress, such as overeating and sedentary behaviour, which contribute to weight gain [30]. Applying this theory, we hypothesise that socioeconomic background moderates the relationship between schoolwork pressure and adolescent obesity, with children from lower socioeconomic backgrounds being more vulnerable to the adverse health effects of schoolwork pressure.

In sum, this study aims to investigate the relationship between schoolwork pressure and overweight/obesity among 5th (≈11 years old), 7th (≈13 years old), and 9th (≈15 years old) grade schoolchildren in Sweden. A secondary aim is to examine whether this association is moderated by socioeconomic background. Finally, assuming schoolwork pressure is a true risk factor for overweight and obesity, we quantified the contribution of schoolwork pressure to the prevalence of overweight/obesity.

## 2 Methods

### 2.1 Design, Sampling, and Data Collection

This study analyses secondary data from the Swedish component of the Health Behaviour in School‐Aged Children (HBSC) survey, a nationally representative sample, school‐based questionnaire administered every 4 years to students in grades 5 (≈11 years old), 7 (≈13 years old), and 9 (≈15 years old) [31]. The survey employed a two‐stage cluster sampling method, with the primary sampling unit being the school class. In the first stage, schools were randomly selected from various regions to ensure the representativeness of the school‐aged population in Sweden. In the second stage, one class from each selected school was chosen, and all students within that class were invited to participate in the survey. The priority for sampling is based on age and the survey is handed out to students in the different grades to produce samples with desired mean ages of 11.5, 13.5, and 15.5 (Table S1). The students fill out the survey in the classroom during school time. This approach was selected to efficiently capture a diverse and representative sample while minimising logistical challenges [32].

The present study uses the results of the three latest HBSC surveys from 2013/14, 2017/18, and 2021/22. The total sample size from the three latest survey rounds was 16 728. Inclusion and exclusion criteria impacted the final sample size (Table S2). Following listwise deletion, cases with no missing data on relevant variables were included, resulting in varying sample sizes across models. Descriptive statistics for both the original and final samples confirmed representativeness after exclusions (Table S3).

### 2.2 Ethical Considerations

The study was conducted in accordance, with local legislation and institutional guidelines (Swedish Ethical Review Board: https://etikprovningsmyndigheten.se/en/what‐the‐act‐says/) and in accordance with the Declaration of Helsinki. Participants were informed about the purpose of the study and data storage and security. While participation in the survey was both anonymous and voluntary, written informed consent was obtained directly from the students and parents/guardians. Parents/guardians who did not wish for their children to participate were asked to notify the school. The Public Health Agency of Sweden and Statistics Sweden (SCB) comply with the EU's data protection regulation (General Data Protection Regulation, GDPR). For this study, a data request for variables from HBSC via the Public Health Agency of Sweden was submitted on January 30th, 2024, then accessed on February 6th, 2024, and thereafter. The data file was converted from SPSS to STATA. Only anonymized secondary data was used, requiring no additional permissions beyond HBSC's approval from the Swedish Ethical Review Board through the Public Health Agency of Sweden.

### 2.3 Measures

#### 2.3.1 Exposure Variable

*Schoolwork pressure*, is measured by responses to the following question: “How pressured do you feel about the schoolwork you have to do?” The question is asked to all age categories, and the students can either answer 1 = not at all, 2 = a little, 3 = some, or 4 = a lot. This variable has been part of HBSC since the 1997/98 survey round [3]. Following the conventions of previous Swedish and international reports from the HBSC network [4, 33], responses were dichotomized into feeling some/a lot of pressure (options 3 and 4) versus feeling not at all/little pressure (options 1 and 2).

#### 2.3.2 Outcome Variable

*Overweight and obesity* are based on body mass index (BMI) calculations using self‐reported height and weight measurements from the survey. The BMI weight status categories were classified using the International Task Force on Obesity's (IOTF) age and sex‐standardised growth curve [34]. The respondents are categorised into different weight categories, including thinness, normal weight, overweight, or obesity, based on age‐specific (in months) and gender‐specific BMI cut‐offs. The BMI cut‐offs in the IOTF growth curve correspond to the adult BMI thresholds of 25 kg/m^2^ (overweight) and 30 kg/m^2^ (obesity) at age 18. In the present study, the categories of thinness and normal weight were combined into a single category. Subsequently, this classification was recoded to distinguish between adolescents with no overweight or obesity (equivalent to BMI < 25 at age 18) and those classified as having overweight, including obesity (equivalent to BMI ≥ 25 at age 18). To assess robustness, the prevalence of overweight/obesity was compared using both IOTF and WHO cut‐offs [35] (Tables S4 and S5).

#### 2.3.3 Sociodemographic Variables

*Gender* is measured as either boy, girl, or other in the HBSC survey. Given the significantly lower frequency for “other” gender (0.67%) in the data sample, that it was only available starting in the 2021/22 survey year [3], and that the IOTF calculations apply to the gender binary [34], this study only included respondents who identify as a boy or a girl. *Age* is reflected by the categorical variable school grade, where grade 5 (≈11 years old), grade 7 (≈13 years old) and grade 9 (≈15 years old).

*Family structure* is measured through several items. Respondents are first asked to: “Please answer the question for the home where you live most of the time and tick the people who live there.” Then, follow‐up questions ask the respondents if they live in an additional home, how often they live there (half of the time, regularly but less than half, sometimes, or rarely), and who lives in that home. The family structure question has been validated in HBSC studies, which have found associations between the variable and adolescent health outcomes [4, 36]. Those living with their mother and father in one home were categorised as a nuclear family. Individuals living in two homes and splitting time between them equally were categorised as alternating. Those living in two homes but spending less than half of their time in the second home were categorised as sometimes. Living with one parent in one home was categorised as a single household. Lastly, adolescents living with neither parent were classified as none.

*Socioeconomic background* was assessed using two variables: an objective measure, Family Affluence Scale (FAS) group, and a subjective measure, Perceived Family Wealth.

*The Family Affluence Scale* has been validated to measure material assets and lifestyle in Sweden [37]. There have been three scale versions so far; this study employed the latest version, FAS‐III, which was developed in 2013/14 [38]. Responses to the six FAS questions were summed and transformed into age‐group and gender‐specific ridit scores. The ridit scores subsequently divided respondents into quintiles which were collapsed into three categories according to guidelines from the HBSC network: [38] the bottom 20% (low FAS), the middle 60% (medium FAS), and the top 20% (high FAS).

*Perceived Family Wealth*, is a relative measure of family wealth, assessed through the responses to the survey question: “How well off do you think your family is?” The students can either answer 1 = very well off, 2 = quite well off, 3 = average, 4 = not so well off, or 5 = not at all well off. The variable was recoded into a categorical variable with three categories, where 1 and 2 from the original dataset were coded as well off, 3 remained as average, and 4 and 5 as not well off. The measure has been included in the HBSC study since 1993/94 and has since been well‐functioning and utilised in Sweden when investigating socioeconomic differences in adolescent health [39].

### 2.4 Statistical Analyses

First, descriptive statistics were generated for all variables to assess distribution and response frequencies. Multicollinearity was assessed using two‐way frequency tables and chi‐square tests. The correlation between the ordinal variables FAS and perceived family wealth variables was assessed using Spearman's rank correlation. Given the low Spearman's rank correlation coefficient [40] (*ρ* = 0.23), there was no need to run separate models for the FAS and perceived family wealth variables.

Then, a series of logistic regression analyses were performed to investigate the associations between schoolwork pressure and overweight/obesity. Four models were run with results presented as odds ratios (OR) with 95% confidence intervals (CI). First, the associations for each covariate with overweight/obesity (outcome) were estimated separately in crude analyses. Then, model 1 included schoolwork pressure (exposure) adjusted for gender, age, and family structure. This was followed by model 2, which was adjusted for all covariates (schoolwork pressure, gender, age, family structure, FAS, and perceived family wealth). Model 3 analysed the potential interaction between schoolwork pressure and FAS, and model 4 explored the interaction between schoolwork pressure and perceived family wealth. Lastly, population attributable fractions (PAF) were calculated to quantify the extent to which the experience of high levels of schoolwork pressure contributes to overweight and obesity, assuming a causal relationship. PAF was calculated in three steps. First, with a crude model, then adjusted for gender, age, and family structure, and lastly adjusting for all covariates including FAS and PFW. Results are reported with 95% CI.

All statistical analyses were performed using Stata/Standard Edition (SE) 18.0 (StataCorp, College Station, TX, USA).

## 3 Results

### 3.1 Descriptive Characteristics

The sample consisted of 5738 respondents from the 2013/14 survey year, 3311 from 2017/18, and 3105 from 2021/22 (Table 1). For all survey years, the gender composition is well balanced for boys and girls. The school grade distribution appears to be similar across the survey years and compared to each other. Most students live in one home with two parents across all survey years. The proportion of missing data for the family structure variable is similar, between 10% and 11%, for all survey years. There is a greater proportion of adolescents with high FAS compared to low FAS in 2013/14 and 2017/18, while, in 2021/22, there is a greater proportion in low FAS. For perceived family wealth, over 80% identify as well‐off, about 15% as average, and 2%–3% as not well‐off across all survey years.

**TABLE 1:** Descriptive statistics of the study sample by survey year and total.

	2013/14		2017/18		2021/22		All	
	*n*	%	*n*	%	*n*	%	*n*	%
Gender								
Boys	2808	48.9	1643	49.6	1622	52.2	6073	50.0
Girls	2930	51.1	1668	50.4	1483	47.7	6081	50.0
Grade								
5th	1938	33.8	848	25.6	1024	33.0	3810	31.4
7th	1745	30.4	1106	33.4	1288	41.5	4139	34.0
9th	2034	35.4	1357	41.0	793	25.5	4184	34.4
Missing	21	0.4	—	—	—	—	21	0.2
Family Structure								
Nuclear	3761	64.0	2171	65.6	2128	68.5	7970	65.6
Sometimes	489	8.5	248	7.5	203	6.5	940	7.7
Alternating	484	8.4	186	5.6	156	5.0	826	6.8
Single household	376	6.6	227	6.9	185	6.0	788	6.5
No parents	67	1.2	137	4.1	100	3.3	304	2.5
Missing	651	11.3	342	10.3	333	10.7	1326	10.9
Family Affluence Scale								
Low	859	15.0	433	13.1	637	20.5	1929	15.9
Medium	3181	55.4	1802	54.4	1899	61.2	6882	56.6
High	1392	24.3	979	29.6	494	15.9	2865	23.6
Missing	306	5.3	97	2.9	75	2.4	478	3.9
Perceived family wealth								
Not well‐off	181	3.1	76	2.3	67	2.2	324	2.7
Average	847	14.8	481	14.5	461	14.8	1789	14.7
Well‐off	4604	80.2	2704	81.7	2.55	82.3	9863	81.1
Schoolwork pressure								
Not at all/a little	4031	70.2	1930	58.3	1699	54.7	7660	63.0
Some/a lot	1707	29.8	1381	41.7	1406	45.3	4494	37.0
Weight status (IOTF)								
No overweight or obesity	4953	86.3	2862	86.4	2649	85.3	10 464	86.1
Overweight, including obesity	785	13.7	449	13.6	456	14.7	1690	13.9
Weight status (WHO)								
No overweight or obesity	4712	82.1	2705	81.7	2512	80.9	9929	81.7
Overweight, including obesity	1026	17.9	606	18.3	593	19.1	2225	18.3
Total	5738	100.0	3311	100.0	3105	100.0	12 154	100.0

Abbreviations: IOTF: International Obesity Task Force; WHO: World Health Organization.

### 3.2 Associations Between Schoolwork Pressure and Overweight/Obesity

Table 2 presents the results of the logistic models. The crude analysis showed a positive association between some/a lot of schoolwork pressure and overweight or obesity (OR 1.17, 95% CI 1.05–1.30). This pattern is noticeable among 7th (OR 1.20, 95% CI 1.05–1.26) and 9th graders (OR 1.36, 95% CI 1.20–1.55), adolescents alternating between two homes (OR 1.38, 95% CI 1.14–1.67), living in single‐parent households (OR 1.57, 95% CI 1.30–1.90), and with no parents (OR 1.36, 95% CI 1.00–1.84), compared to the reference categories. The odds of overweight/obesity were lower among girls (OR 0.66, 95% CI 0.59–0.73) than boys. Medium FAS (OR 1.31, 95% CI 1.14–1.50), low FAS (OR 1.98, 95% CI 1.68–2.34), average perceived family wealth (OR 1.38, 95% CI 1.20–1.58), and not well‐off perceived family wealth (OR 2.07, 95% CI 1.59–2.68) were positively associated with overweight/obesity in the crude analysis.

**TABLE 2:** Results from logistic regression analysis of overweight/obesity regressed on schoolwork pressure and covariates.

Variables	Crude		Model 1		Model 2		Model 3		Model 4	
	OR	95% CI	OR	95% CI	OR	95% CI	OR	95% CI	OR	95% CI
		Overweight/obesity		Overweight/obesity		Overweight/obesity		Overweight/obesity		Overweight/obesity
Schoolwork pressure										
Not at all/a little (ref.)	1.00		1.00		1.00					
Some/a lot	1.17**	[1.05–1.30]	1.17*	[1.04–1.33]	1.17*	[1.03–1.32]				
Survey year										
2013/14 (ref.)	1.00		1.00		1.00		1.00		1.00	
2017/2018	0.99	[0.87–1.12]	0.98	[0.86–1.13]	1.001	[0.87–1.15]	1.001	[0.87–1.15]	1.001	[0.87–1.15]
2021/2022	1.09	[0.96–1.23]	1.09	[0.95–1.25]	1.03	[0.90–1.19]	1.03	[0.90–1.19]	1.03	[0.90–1.19]
Gender										
Boy (ref.)	1.00		1.00		1.00		1.00		1.00	
Girl	0.66**	[0.59–0.73]	0.64**	[0.57–0.72]	0.63**	[0.56–0.71]	0.63**	[0.56–0.71]	0.63**	[0.56–0.71]
Grades										
5th (ref.)	1.00		1.00		1.00		1.00		1.00	
7th	1.20**	[1.05–1.36]	1.19*	[1.03–1.37]	1.19*	[1.03–1.38]	1.19*	[1.03–1.38]	1.19*	[1.03–1.38]
9th	1.36**	[1.20–1.55]	1.32**	[1.14–1.52]	1.32**	[1.13–1.54]	1.32**	[1.13–1.54]	1.32**	[1.13–1.54]
Family structure										
Nuclear (ref.)	1.00		1.00		1.00		1.00		1.00	
Sometimes	1.004	[0.82–1.23]	1.01	[0.82–1.23]	0.99	[0.80–1.22]	0.99	[0.80–1.22]	0.99	[0.80–1.22]
Alternating	1.38*	[1.14–1.67]	1.37**	[1.13–1.67]	1.22	[0.99–1.50]	1.22	[0.99–1.50]	1.22	[0.99–1.50]
Single	1.57**	[1.30–1.90]	1.53**	[1.26–1.85]	1.30*	[1.06–1.60]	1.30*	[1.07–1.60]	1.30*	[1.06–1.60]
None	1.36*	[1.00–1.84]	1.32	[0.97–1.80]	1.32	[0.95–1.84]	1.32	[0.95–1.84]	1.32	[0.95–1.84]
FAS										
High (ref.)	1.00				1.00				1.00	
Low	1.98**	[1.68–2.34]			1.85**	[1.53–2.24]			1.85**	[1.53–2.24]
Medium	1.31**	[1.14–1.50]			1.37**	[1.18–1.59]			1.37**	[1.18–1.59]
Perceived family wealth										
Well‐off (ref.)	1.00				1.00		1.00			
Not well‐off	2.07**	[1.59–2.68]			1.62**	[1.20–2.19]	1.63**	[1.20–2.21]		
Average	1.38**	[1.20–1.58]			1.10	[0.94–1.29]	1.10	[0.94–1.29]		
Schoolwork pressure × FAS										
Not at all/a little × High (ref.)							1.00			
Not at all/a little × Low							2.00**	[1.57–2.54]		
Not at all/a little × Medium							1.41**	[1.16–1.71]		
Some/a lot × High							1.26	[0.97–1.65]		
Some/a lot × Low							2.10**	[1.60–2.76]		
Some/a lot × Medium							1.66**	[1.34–2.06]		
Schoolwork pressure × Perceived family wealth										
Not at all/a little × Well off (ref.)									1.00	
Not at all/a little × Not well off									1.71*	[1.09–2.66]
Not at all/a little × Average									1.10	[0.89–1.37]
Some/a lot × Well off									1.17*	[1.02–1.35]
Some/a lot × Not well off									1.82**	[1.22–2.72]
Some/a lot × Average									1.29*	[1.03–1.61]
Intercept	0.15**	[0.14–0.16]	0.15**	[0.13–0.17]	0.12**	[0.09–0.13]	0.12**	[0.09–0.13]	0.11**	[0.09–0.13]
Wald test							*χ* ^2^ = 46.3	*p* < 0.000	*χ* ^*2*^ = 16.9	*p* = 0.0047
Number of observations	12 154		10 811		10 339		10 339		10 339	

*Note:* Crude analyses examined bivariate associations.

Abbreviations: CI: confidence interval; OR: odds ratio; Ref: reference group.

* *p* < 0.05.

** *p* < 0.01.

Schoolwork pressure remained statistically significantly associated with the prevalence of overweight or obesity when controlling for all covariates.

The possible moderating effect of socioeconomic background on the association between schoolwork pressure and weight status was explored by conducting interaction analyses. The interaction between schoolwork pressure and FAS was investigated in model 3, and that between schoolwork pressure and perceived family wealth was investigated in model 4. As indicated by the Wald test, the overall interaction terms were statistically significant between schoolwork pressure and FAS (*χ* ^2^ = 46.3 *p* < 0.001) and between schoolwork pressure and perceived family wealth (*χ* ^2^ = 16.9 *p* = 0.005).

The findings indicate a significant interaction between schoolwork pressure and family affluence on the odds of overweight/obesity. Adolescents with low family affluence and some or a lot of schoolwork pressure had the highest odds of overweight/obesity (OR 2.10, 95% CI 1.60–2.76) compared to those with high FAS and low schoolwork pressure. Similarly, students with medium FAS and high schoolwork pressure had significantly elevated odds (OR 1.66, 95% CI 1.34–2.06) compared to the reference. Even with low schoolwork pressure, students with low FAS faced higher odds (OR 2.00, 95% CI 1.57–2.54). For students with medium FAS and low schoolwork pressure, the odds of overweight/obesity were 1.41 times higher (95% CI 1.16–2.71) compared to students with high FAS and little to no schoolwork pressure. No statistically significant difference was observed between high FAS groups with differing schoolwork pressure levels.

A statistically significant interaction was also found between schoolwork pressure and perceived family wealth. Students from not well‐off families with moderate/high schoolwork pressure had higher odds of overweight/obesity (OR 1.82, 95% CI 1.22–2.71) compared to those with low schoolwork pressure and well‐off families. Adolescents with average perceived wealth and moderate/high schoolwork pressure also had higher odds of overweight, including obesity (OR 1.29, 95% CI 1.03–1.61). The odds of overweight/obesity were higher for students who experienced little to no schoolwork pressure and were from not well‐off families (OR 1.71, 95% CI 1.09–2.66) as compared to those with the same pressure levels but from a well‐off family. Additionally, students from well‐off families with moderate/high schoolwork pressure had elevated odds of having overweight and obesity (OR 1.17, 95% CI 1.02–1.35). However, no significant effect was found for average wealth combined with low schoolwork pressure.

### 3.3 Population Attributable Fraction

The study also quantified the extent to which the experience of high levels of schoolwork pressure contributes to the prevalence of overweight and obesity using PAF. The PAF calculations assume that schoolwork pressure is a true risk factor for overweight/obesity. The overall prevalence of overweight, including obesity in the study population was about 14%. The PAF calculations indicated that about 5.1% of the cases of overweight/obesity were attributable to high levels of schoolwork pressure (Table 3) when not controlling for other covariates (95% CI 0.02–0.09). The PAF did not change when controlling for covariates as in model 1 (gender, age, and family structure). When adding in FAS and perceived family wealth, as in model 2, the PAF decreased slightly to 4.9% (95% CI 0.01–0.09).

**TABLE 3:** Population attributable fraction for crude analysis and adjusted models 1 and 2, with associated 95% CI.

	PAF estimate	95% CI
Crude	0.051	0.02–0.09
Model 1	0.051	0.01–0.09
Model 2	0.049	0.01–0.09

Abbreviations: CI: confidence interval; PAF: population attributable fraction.

## 4 Discussion

This study used nationally representative pooled cross‐sectional data to investigate the association between schoolwork pressure and overweight/obesity among Swedish children and adolescents in grades 5, 7 and 9. Then, the potential moderating effect of socioeconomic background, as well as gender, age, and family structure differences in the odds of having overweight or obesity, was explored. Lastly, population attributable fractions were calculated to quantify schoolwork pressure's contribution to overweight/obesity.

The results of the current study indicated that the proportion of students experiencing some or a lot of schoolwork pressure has in over time. In the 2021/22 school year, approximately half of the students reported experiencing some or a lot of pressure, marking a 15% increase from the 2013/14 school year. This rise may partially be attributed to the changes in Sweden's grading system in 2011 [2], which introduced more testing and stricter entrance requirements to high school programmes. This is in line with the results of at least one Swedish study which found that higher school demands were associated with increased stress in 14–16‐year‐olds [41]. Gender differences were also noted in the current study as girls were more likely than boys to experience high schoolwork pressure, consistent with prior research on Swedish adolescents [6, 41]. However, overweight and obesity were more prevalent among boys than girls. These gender differences may reflect social, behavioural, and biological factors [5]. For example, societal norms around body image, differing coping strategies, and variations in stress physiology may help explain these divergent outcomes [5].

In analyses that explored the relationship between schoolwork pressure and overweight/obesity, we found that approximately 37% of students reported high schoolwork pressure, while 14% of all students were categorised as having overweight or obesity. The results showed that greater levels of schoolwork pressure were associated with 17% higher odds of overweight or obesity. The association remained statistically significant even after adjusting for potential confounders, including gender, age, family structure, FAS, and perceived family wealth. These findings are consistent with prior research suggesting a link between academic stress and weight status [12, 18, 42]. For example, a Greek study on 10–12‐year‐olds found that academic stress was linked to higher odds of overweight and obesity, even after controlling for gender and age [18]. Additionally, evidence from a study of Bangladeshi adolescents aged 13–19 also supports this relationship, showing that perceived stress, including stress from future uncertainty and school performance, was positively correlated with higher BMI and unhealthy eating behaviours, such as emotional eating and eating in response to external cues [12]. The observed association in the present study may be interpreted through the lens of Lazarus and Folkman's transactional model of stress and coping, which posits that when perceived demands, such as pressure related to schoolwork, exceed coping resources, individuals experience psychological strain and may adopt emotion‐focused coping strategies such as stress‐related eating [43]. Notably, the relationship between school‐related stress and overweight/obesity is not limited to childhood and adolescence. A similar association was found among university students [42], suggesting that the association between academic stress on weight status may persist into young adulthood. Chronic exposure to such stressors has been proposed to activate physiological pathways, particularly the hypothalamic–pituitary–adrenal (HPA) axis, potentially leading to elevated cortisol levels that promote appetite and fat accumulation [44].

A secondary aim of our study was to determine if the association between schoolwork pressure and overweight/obesity varies across different socioeconomic groups. The overall interaction terms between schoolwork pressure and FAS and between schoolwork pressure and perceived family wealth were statistically significant. These results suggest that the association between schoolwork pressure and overweight/obesity varies depending on socioeconomic background. The likelihood of having overweight or obesity was greatest among those experiencing some or a lot of schoolwork pressure who also had poor socioeconomic backgrounds (i.e., low FAS or low perceived family wealth). These findings are consistent with the framework proposed by Hemmingsson [30], where the existence of adverse socioeconomic conditions in the home may heighten the experience of stressors and lead to a greater risk of developing overweight and obesity.

The results also revealed that adolescents from families with low FAS and those perceiving their family as less well‐off were more likely to have overweight or obesity compared to adolescents from higher socioeconomic backgrounds, even when facing little to no schoolwork pressure. It seems that poorer socioeconomic conditions may be a risk factor for overweight or obesity regardless of schoolwork pressure level. This finding suggests that an adverse socioeconomic background is a stressor in and of itself, increasing the odds of overweight/obesity [25, 26, 27], as argued in Hemmingsson's model [30]. Adolescents from lower socioeconomic backgrounds may face barriers to accessing healthy foods at home and engaging in structured physical activity [16] or model unhealthy behaviours exhibited by their parents [45], contributing to socioeconomic disparities in the prevalence of overweight/obesity.

Furthermore, adolescents who perceive their families as well‐off and experiencing more schoolwork pressure have 17% higher odds of overweight/obesity compared to their peers with little to no schoolwork pressure. In contrast, adolescents who perceive their families as not well‐off and experience a lot of schoolwork pressure have 82% higher odds than those with little to no pressure and well‐off families, suggesting that perceived affluence may protect against schoolwork pressure's impact on overweight/obesity. One explanation is that adolescents from wealthier families may have greater access to health‐promoting resources [16] and stronger support systems that help mitigate the effects of stress [13]. Notably, this interaction effect emerged only when using perceived family wealth as a measure of socioeconomic background, not FAS, to measure socioeconomic states. This distinction underscores the value of assessing both objective (FAS) and subjective (perceived wealth) socioeconomic indicators, as they may relate differently to health outcomes [39]. However, because adolescents reported perceived wealth and schoolwork pressure simultaneously, there is a potential endogeneity issue where those who feel their family is less well‐off may also perceive greater stress, making it difficult to entangle these effects.

The third aim of the study was to quantify the contribution of high schoolwork pressure to overweight and obesity prevalence using PAF. Assuming schoolwork pressure is a true risk factor, the counterfactual scenario hypothesises that reducing schoolwork pressure could lower overweight/obesity prevalence by about 5%. The PAF results remained largely unchanged after adjusting for age, gender, family structure, or socioeconomic background. Given that the overall prevalence of overweight and obesity in the studied population was about 14%, the contribution of schoolwork pressure to this burden is notable. Previous research has used PAF to quantify the burden of specific risk factors on childhood overweight/obesity [46]. A US study found that nearly 35% of obesity cases among 9–12th graders could be attributed to modifiable risk factors, including, but not limited to, unhealthy dietary behaviours, physical activity, and screen time usage [46]. These factors have been previously shown to be associated with overweight/obesity [12, 47] and stress [11]. Given that the current study is based on cross‐sectional data, it is important to note that the PAF linked to schoolwork pressure and the prevalence of overweight/obesity reflects an association rather than a causal relationship.

### 4.1 Public Health Implications and Future Recommendations

The observations of this study highlight how efforts to mitigate excessive schoolwork pressure could contribute to a reduction in the prevalence of overweight and obesity among Swedish adolescents. This, in turn, may reduce a wide range of short and long‐term economic, social, and public health issues. As previously noted, Swedish adolescents have reported increasing levels of perceived schoolwork pressure across all age groups and among both boys and girls [3]. In light of Sweden's 2011 shift to a more competitive grading system, policymakers should monitor and address academic pressure as a potential health risk. Addressing this requires multi‐level interventions based on the Social Ecological Model [29]. Combining individual strategies, such as stress management skills, with efforts to create supportive and less stressful school environments may be effective. Research has shown that strong school‐based coping resources are linked to reduced stress levels [41]. These findings indicate that enhancing school environments and support systems may help to alleviate school‐related stress.

In addition, future research should explore how food environments, alongside school settings, influence the relationship between schoolwork pressure and adolescent weight status. Food environments, including availability, accessibility, and marketing, significantly contribute to socioeconomic disparities in eating behaviours and weight [48]. Children and adolescents are particularly affected by food advertisements, which can lead to unhealthy eating habits [49]. Hemmingsson's model suggests that stress in disadvantaged socioeconomic conditions fosters maladaptive coping mechanisms, such as obesogenic eating behaviours [30]. Additionally, research indicates that supermarkets in lower socioeconomic areas of Sweden advertise more unhealthy foods [50]. Understanding these factors is crucial for mitigating the effects of schoolwork pressure on adolescent weight.

### 4.2 Strengths and Limitations

To the best of our knowledge, this is the first study to examine the association between schoolwork pressure and overweight/obesity in Sweden using a nationally representative multi‐year dataset. Previous studies in Sweden have mainly focused on the effects of school‐related stress on psychosomatic and mental health outcomes. However, the present study adds a new dimension to the detrimental effects schoolwork pressure may have on adolescent health. By including recent survey years, the trends and associations are based on up‐to‐date data. Validated measures widely used in international and Swedish adolescent health research were employed [33, 37, 39], including BMI which was categorised by IOTF growth curves [34].

A number of limitations of this study should be mentioned. Firstly, measures were taken to address confounding factors such as gender, age, family structure, and socioeconomic background, but there may be other influential variables, such as physical activity and eating behaviours. Previous studies suggest that physical activity does not change the association between schoolwork pressure and weight status [12, 18], though eating behaviours may play a significant role [10]. Secondly, as this study relied on self‐reported cross‐sectional data, causality cannot be established. Additionally, the cross‐sectional design prevents determining the direction of the relationship. Lastly, using self‐reported weight and height data introduces some risk of misclassification [51, 52]. Research indicates that girls are more likely to underestimate their weight, while boys are more likely to overestimate their height [51]. Overall, self‐reported data tends to underestimate the prevalence of overweight/obesity compared to measured data [52]. Consequently, the association found between schoolwork pressure and overweight/obesity in this study may be underestimated rather than the other way around.

## 5 Conclusions

This study demonstrates a positive association between schoolwork pressure and overweight/obesity among Swedish adolescents, even after adjusting for key sociodemographic factors. The findings suggested that stress related to academic work, a growing concern among adolescent populations, may be a critical yet underrecognised factor contributing to the rising burden of overweight and obesity. Importantly, adolescents from lower socioeconomic backgrounds not only experienced higher baseline prevalence of overweight and obesity but were also more vulnerable to the adverse impacts of schoolwork pressure on weight status. These findings underscore the need for public health interventions that not only address academic stress broadly but also provide targeted support for socioeconomically disadvantaged youth.

## Author Contributions

**C.C., K.R.J., and M.C.:** conceptualization. **C.C., K.R.J., and M.C.:** methodology. **C.C. and K.R.J.:** formal analysis. All authors were involved in writing the paper and had final approval of the submitted and published versions.

## Conflicts of Interest

The authors declare no conflicts of interest.

## Supporting information


**Data S1:** Supporting Information.

## Data Availability

Application and enquiries for data use should be sent to skolbarns.hälsovanor@fohm.se.
