# Supplementary material for: The Association Between Schoolwork Pressure and Overweight/Obesity in Swedish Schoolchildren Across Different Socioeconomic Groups
Source: Pediatr Obes. 2025 Nov 12;21(1):e70067. doi: 10.1111/ijpo.70067 (PMC12696444; doi:10.1111/ijpo.70067)
Supplement: Supplementary file 1 — Data S1: Supporting Information. [file IJPO-21-e70067-s001.pdf]

The association between schoolwork pressure and overweight/obesity in Swedish schoolchildren across different socioeconomic groups

Cassandra Comey
School of Public Health and Community Medicine, Sahlgrenska Academy University of Gothenburg, Gothenburg, Sweden
cassandra.comey@gu.se
ORCID: 0009-0007-8683-7336

Kenisha Russell Jonsson
School of Public Health and Community Medicine, Sahlgrenska Academy University of Gothenburg, Gothenburg, Sweden
kenisha.russell.jonsson@gu.se
ORCID: 0000-0002-7835-4592

Maria Corell
School of Public Health and Community Medicine, Sahlgrenska Academy University of Gothenburg, Gothenburg, Sweden
maria.corell@gu.se
ORCID 0000-0001-8591-0107

Corresponding author:
Cassandra Comey
School of Public Health and Community Medicine, Sahlgrenska Academy University of Gothenburg, Gothenburg, Sweden
cassandra.comey@gu.se

Key words:
Children and adolescents, overweight, obesity, schoolwork pressure, socioeconomic status

Supplementary information

Table S1. Number of participating students and schools with response rates at school and student level.

Survey year	Schools sampled (N)	Participating schools (N)	Response rate among schools	Participating students (N)	Response rate among students	Time of data collection
2013/14	500	386	77%	7,867	90%	January 2014-March 2014
2017/18	450	213	47%	4,294	89%	October 2017-January 2018
2021/22	450	247	55%	4,567	80%	October 2021-May 2022

Table S2. Total respondents, exclusion criteria, and missing data

Variable	n	%
**All HBSC respondents** (2013/14, 2017/18, 2021/22)	**16,728**	**100**
Excluded respondents due to missing weight and height measurements	2,542	15.2
Excluded respondents where height > weight	161	0.01
Excluded respondents where 5 > BMI z-scores < -5	360	0.02
Excluded respondents where BMI > 50	190	0.01
Excluded respondents due to missing gender or “other” gender	198	0.01
Excluded respondents where 16 > age < 10	931	0.06
Excluded respondents due to missing response to schoolwork pressure question	192	0.01
**Full sample for analysis**	**12,154**	**100**
**N without missing value**	10,339	85.1
**Variables with missing value**:		
Grade	21	0.2
Family structure	1,326	10.9
Family Affluence Scale (FAS)	478	3.9

HBSC: Health Behaviour in School-aged Children study; BMI: Body Mass Index.

Table S3. Sociodemographic characteristics of covariates (gender, grade, FAS, perceived family wealth, and family structure) in the study population, prior to the exclusion of data, by survey year and total.

	2013/14		2017/18		2021/22		All	
	n	%	n	%	n	%	n	%
**Gender**								
Boys	3,896	49.5	2,101	48.9	2,224	48.7	8,221	49.2
Girls	3,908	49.7	2,114	49.2	2,191	48.0	8,213	49.1
Other					107	2.3	107	0.6
Missing	63	0.8	79	1.9	45	1.0	187	1.1
Total	7,867	100.0	4,294	100.0	4,567	100.0	16,728	100.0
**Grade**								
5th	2,691	34.2	1,181	27.5	1,559	34.1	5,431	32.5
7th	2,292	29.1	1,452	33.8	1,575	34.5	5,319	31.8
9th	2,810	35.7	1,661	38.7	1,433	31.4	5,904	35.3
Missing	74	0.9					74	0.4
Total	7,867	100.0	4,294	100.0	4,567	100.0	16,728	100.0
**Family Affluence Scale**								
Low	1,294	16.4	657	15.3	978	21.4	2,929	17.5
Medium	4,270	54.3	2,284	53.2	2,725	59.7	9,279	55.5
High	1,728	22.7	1,170	27.2	691	15.1	3,647	21.8
Missing	517	6.6	183	4.3	173	3.8	873	5.2
Total	7,867	100.0	4,294	100.0	4,567	100.0	16,728	100.0
**Perceived family wealth**								
Not well off	273	3.5	109	2.5	110	2.4	492	2.9
Average	1,140	14.5	627	14.6	675	14.8	2,442	14.6
Well off	6,246	79.4	3,453	80.4	3,706	81.1	13,405	80.2
Missing	208	2.6	105	2.5	76	1.7	389	2.3
Total	7,867	100.0	4,294	100.0	4,567	100.0	16,728	100.0
**Family Structure**								
Nuclear	4,833	61.4	2,741	63.8	3,066	67.1	10,640	63.6
Sometimes	631	8.0	295	6.9	284	6.2	1,210	7.2
Alternating	674	8.6	241	5.6	231	5.1	1,146	6.9
Single household	570	7.3	333	7.8	294	6.5	1,197	7.1
No parents	148	1.9	211	4.9	184	4.0	543	3.3
Missing	1,011	12.8	473	11.0	508	11.1	1,992	11.9
Total	7,867	100.0	4,294	100.0	4,567	100.0	16,728	100.0

Table S4. Distribution of weight statuses based on IOTF cut-offs.[41]

BMI	Frequency	Percentage
Grade 3 thinness	124	1.00
Grade 2 thinness	248	2.0
Grade 1 thinness	1,183	9.6
Normal weight	9,070	73.5
Overweight	1,442	11.6
Obese	279	2.3
Total	12,346	100

Table S5. Distribution of weight statuses based on WHO cut-offs.[42]

BMI	Frequency	Percentage
Extra underweight	100	0.9
Underweight	364	2.9
Normal	9,616	77.9
Overweight	1,770	14.3
Obesity	496	4.0
Total	12,346	100

Table S6. Marginal effects of all combinations of schoolwork pressure and FAS, and of schoolwork pressure and perceived family wealth, with associated standard error (SE) and 95% confidence intervals.

	**Margin**	**SE**	**95% CI**
**Schoolwork pressure*FAS**			
not at all/a little*high	0.10	0.01	0.09-0.11
not at all/a little*medium	0.13	0.01	0.12-0.14
not at all/a little*low	0.17	0.01	0.15-0.19
some/a lot*high	0.12	0.01	0.10-0.13
some/a lot*medium	0.15	0.01	0.14-0.16
some/a lot*low	0.19	0.01	0.17-0.22
**Schoolwork pressure*Perceived family wealth**			
not at all/a little*well-off	0.13	0.004	0.12-0.14
not at all/a little*average	0.14	0.01	0.12-0.16
not at all/a little*not well-off	0.19	0.02	0.14-0.24
some/a lot*well-off	0.15	0.01	0.13-0.16
some/a lot*average	0.16	0.01	0.14-0.18
some/a lot*not well-off	0.21	0.03	0.16-0.26

Figure S1. A) Interaction plot between schoolwork pressure and perceived family wealth on the probability of overweight and obesity using marginal effects. B) Interaction plot between schoolwork pressure and FAS on the probability of overweight and obesity using marginal effects. With associated confidence intervals.

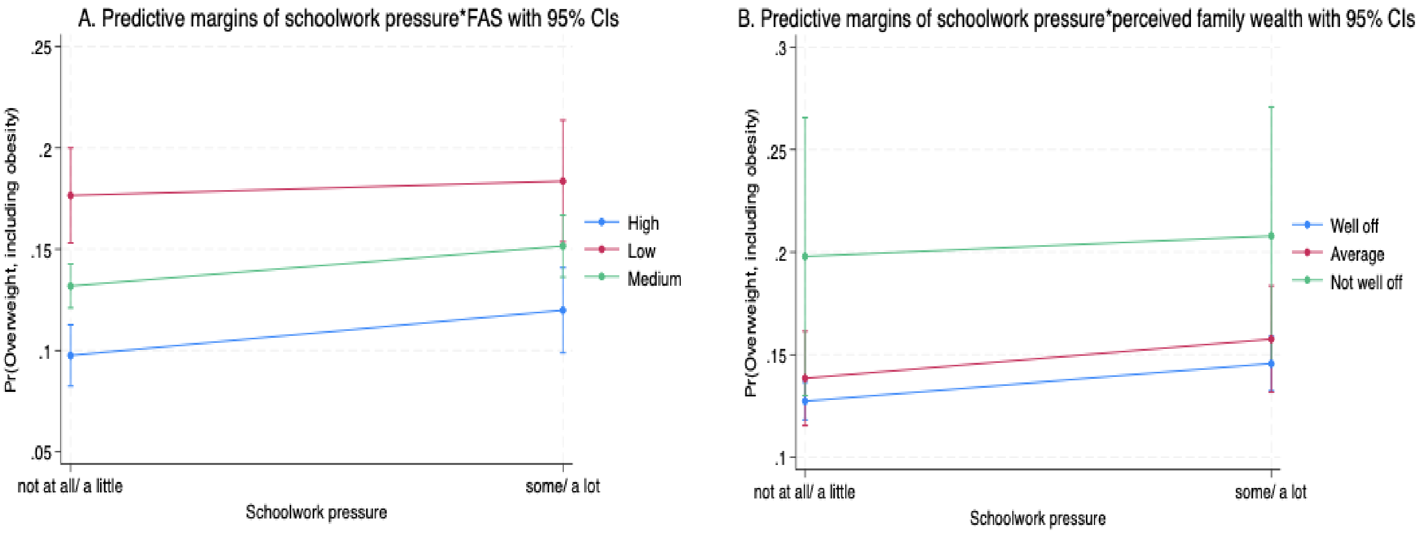

FAS: Family Affluence Scale; CI: Confidence Intervals
